# Pseudospin Dependent One-Way Transmission in Graphene-Based Topological Plasmonic Crystals

**DOI:** 10.1186/s11671-018-2538-x

**Published:** 2018-04-20

**Authors:** Pingping Qiu, Weibin Qiu, Junbo Ren, Zhili Lin, Zeyu Wang, Jia-Xian Wang, Qiang Kan, Jiao-Qing Pan

**Affiliations:** 1 Fujian Key Laboratory of Light Propagation and Transformation, College of Information Science and Engineering, Huaqiao University, Xiamen, 361021 China; 2 College of Materials Science and Opto-Electronic Technology, University of Chinese Academy of Sciences, Beijing, 100086 China; 3 Institute of Semiconductors, Chinese Academy of Sciences, Beijing, 100086 China

**Keywords:** Graphene, Plasmonic crystal, Surface plasmon polaritons, Topological states

## Abstract

Originating from the investigation of condensed matter states, the concept of quantum Hall effect and quantum spin Hall effect (QSHE) has recently been expanded to other field of physics and engineering, e.g., photonics and phononics, giving rise to strikingly unconventional edge modes immune to scattering. Here, we present the plasmonic analog of QSHE in graphene plasmonic crystal (GPC) in mid-infrared frequencies. The band inversion occurs when deforming the honeycomb lattice GPCs, which further leads to the topological band gaps and pseudospin features of the edge states. By overlapping the band gaps with different topologies, we numerically simulated the pseudospin-dependent one-way propagation of edge states. The designed GPC may find potential applications in the fields of topological plasmonics and trigger the exploration of the technique of the pseudospin multiplexing in high-density nanophotonic integrated circuits.

## Background

Photonic topological insulators [1–4], optical materials of a nontrivial topological phase that prohibit light transmission in their interiors but allow propagation along their edges, have been studied intensively following the discovery of quantum Hall effect (QHE) in condensed matter. A key manifestation of topological physics is the presence of edge states which are robust against structural defects or local disorders. Particularly, by utilizing the bulk-edge correspondence [5, 6], one may investigate different topological phases by probing edge states or edge topological invariants. In recent years, topological edge states have been predicted and observed in many photonic topological band gap systems, such as gyromagnetic photonic crystals [7–9], bi-anisotropic-based photonic topological insulators [10, 11], coupled waveguide networks [12, 13], and Floquet photonic lattices [14, 15], where various physical mechanisms are proposed to provide topological protection. Notably, a double Dirac cone was opened to obtain a topologically nontrivial band gap in a well-known honeycomb lattice photonic crystal that preserves pseudo time-reversal symmetry, which gives rise to pseudospin-dependent unidirectional transmission of edge states [16, 17]. Besides the photonic systems, pseudospin-dependent edge states in phononic systems have been explored [18–20]. However, the analogy in the plasmonic nanostructures has not yet been reported, which is due to the huge ohmic loss of the plasmons propagating along the traditional plasmonic materials such as Au and Ag.

Surface plasmon polaritons (SPPs) [21], elementary excitations coupled by photons and free-electron oscillations at an interface between a metal and a dielectric, are regarded as a promising physical mechanism to circumvent the diffraction limitation and to advance the miniaturization of the devices. Iurov et al. explored the back action and hybridization of the plasmon modes and found the induced optical polarization by Dirac electrons in graphene [22]. Memmi et al. reported the strong coupling between SPPs and molecular vibrations [23]. While commonly used noble metals such as gold and silver exhibit plasmonic properties mostly in the visible and near-infrared region of the spectrum, graphene has recently emerged as a promising alternative which is able to extend the field of plasmonics to infrared and terahertz (THz) wavelengths. More importantly, in contrast to noble metals, graphene plasmons can be dynamically tuned via electrostatic biasing [24, 25], which enables a new generation of reconfigurable plasmonic devices. Furthermore, SPPs excited in high-quality graphene can reach remarkably long intrinsic relaxation times and provide unprecedented levels of field confinement [26]. These extraordinary properties make graphene an ideal candidate to the all-integrated topological plasmonic components. Very recently, Jin et al. realized the topologically protected one-way edge plasmons in a periodically patterned monolayer graphene, where the band topology of graphene plasmons under a time-reversal-breaking magnetic field was studied in detail [27]. And Pan et al. demonstrated the substantial nonreciprocal behavior at the superlattice junctions under moderate static magnetic fields, leading to the emergence of topologically protected edge states and localized bulk modes [28].

In this work, we theoretically explore the topological properties of two-dimensional (2D) graphene plasmonic crystals (GPCs) constructed by periodically arranged graphene nanodisks. Dirac cones at the Brillouin zone (BZ) corners are folded to a double Dirac cone at the BZ center by utilizing the zone folding mechanism. In order to obtain topological band gaps, we take further deformations on honeycomb lattice. By shrinking or expanding the graphene nanodisks, the double Dirac cone is opened and the band inversion occurs between pseudospin dipole and quadrupole modes, which further leads to topological phase transition between nontrivial and trivial states. Furthermore, one-way propagation of edge states is numerically simulated along an interface constructed by the trivial and nontrivial GPCs, which further demonstrates the pseudospin characteristics and topological robustness of our designed plasmonic crystals.

## Methods

**Fig. 1:**
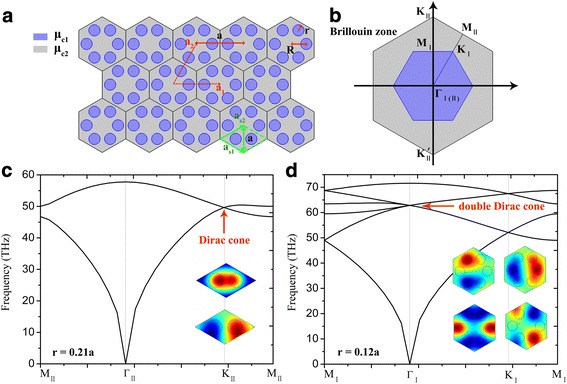
**a** Schematics of the 2D GPCs. **b** The Brillouin zones. **c** Band structure of the lattice based on the rhombic primitive unit cell indicated with green dashed lines, the insets plot the eigen electric field distributions of the Dirac point. **d** Band structure of the lattice based on the hexagonal unit cell, the insets plot the eigen electric field distributions of the double Dirac point. The other parameters are set as *μ*_c1_ = 0.3 eV, *μ*_c2_ = 0.6 eV, *τ* = 1 ps, the lattice constant *a* = 40 nm

We investigate the band topology of SPPs in a 2D plasmonic crystal of an array of periodically arranged graphene nanodisks surrounded by the same sheet of graphene with different chemical potential as shown in Fig. 1a. The lattice constant *a* = 40 nm, *μ*_c1_, and *r* are the chemical potential and radii of the graphene nanodisks; *μ*_c2_ denotes the chemical potential of the surrounding graphene. By solving the Maxwells equations with boundary conditions, we obtain the dispersion relation for transverse magnetic (TM)-polarized SPP modes supported on the graphene layer surrounding by air and silica [29]:

1 $$ \frac{\varepsilon_{\mathrm{Air}}}{\sqrt{\beta^2-{k}_0^2{\varepsilon}_{Air}}}+\frac{\varepsilon_{Si{O}_2}}{\sqrt{\beta^2-{k}_0^2{\varepsilon}_{{\mathrm{SiO}}_2}}}=\frac{\sigma_g}{i{\omega \varepsilon}_0}. $$

Here, *ε*_0_ is the vacuum permittivity of free space, *k*_0_ = 2π/*λ* is the wave number in free space, and *λ* is the operating wavelength in vacuum. In the mid-infrared region, the dielectric constants of air and silica corresponding to super and substrates are assumed to be *ε*_Air_ = 1 and *ε*_SiO2_ = 3.9 respectively [30]. In the non-retarded regime where *β* » *k*_*0*_, the Eq. (3) can be simplified to [31].

2 $$ \beta ={\varepsilon}_0\frac{\varepsilon_{\mathrm{Air}}+{\varepsilon}_{{\mathrm{SiO}}_2}}{2}\frac{2 i\omega}{\sigma_{\mathrm{g}}}, $$

where *β* is the propagation constant SPPs on graphene layer, and the effective refractive index *n*_eff_ of the SPP mode can be derived from *n*_eff_ = *β*/*k*_*0*_. *σ*_g_ is the surface conductivity of graphene composed of the contributions of intraband and interband, i.e., *σ*_g_ = *σ*_intra_ + *σ*_inter_ [29, 30]. The intraband conductivity *σ*_intra_ corresponding to the intraband electron-photon scattering process is given by

3 $$ {\sigma}_{\mathrm{intra}}=\frac{ie^2{k}_BT}{\pi {\mathrm{\hslash}}^2\left(\omega +i/\tau \right)}\left[\frac{\mu_{\mathrm{c}}}{k_BT}+2\ln \left(1+\exp \left(-\frac{\mu_{\mathrm{c}}}{k_BT}\right)\right)\right], $$

where *μ*_c_ is the chemical potential relating to the electron density, *e* is the electron charge, *ω* is the angular frequency of the plasmon, *ℏ* and *k*_*B*_ are the reduced Planck’s constant and the Boltzmann’s constant respectively, *T* is the temperature, and *τ* represents the electron momentum relaxation time due to charge carrier scattering. For *ℏω* » *k*_*B*_*T* and |*μ*_c_| » *k*_*B*_*T*, the interband conductivity *σ*_inter_ corresponding to interband electron transitions can be approximately expressed as

4 $$ {\sigma}_{\mathrm{inter}}=\frac{ie^2}{4\pi \mathrm{\hslash}}\ln \left[\frac{2\mid {\mu}_{\mathrm{c}}\mid -\mathrm{\hslash}\left(\omega +i/\tau \right)}{2\mid {\mu}_{\mathrm{c}}\mid +\mathrm{\hslash}\left(\omega +i/\tau \right)}\right]. $$

## Results and Discussion

The energy band structures of the proposed plasmonic crystals are obtained by employing the finite element method (FEM) based commercially available software COMSOL Multiphysics. In Fig. 1a, we notice that both the rhombic unit cell of two graphene nanodisks (green dashed rhombus defined by vectors **a**_**s1**_ and **a**_**s2**_) and the hexagonal unit cell of six graphene nanodisks (with lattice vectors **a**_**1**_ and **a**_**2**_) can form the honeycomb-lattice plasmonic crystals. Figure 1b presents the BZs for the rhombic and hexagonal unit cells, with the irreducible zones of M_II_-*Γ*_II_- K_II_- M_II_ and M_I_-*Γ*_I_- K_I_- M_I_ respectively. Note that the hexagonal unit cell is three times larger than the rhombic primitive one. Therefore, the first BZ of the rhombic primitive unit cell is three times larger than that of the hexagonal one (blue region in Fig. 1b). When taking a rhombic primitive unit cell, this plasmonic crystal exhibits Dirac cone dispersion at K_II_ and K_II_` points in the BZ corners as shown in Fig. 1c. The insets in Fig. 1c show the eigen electric field distributions of the two degenerated states at Dirac point. Similar to the pseudo-spins in classical photonic and acoustic systems [17, 19, 20], in order to mimic the analog of the pseudo-spins in plasmonic system, the degree of freedom should be increased to twofold states. Thus, fourfold degenerated double Dirac cones in the plasmonic band structure are required. By employing zone folding mechanism [18], the Dirac cones at K_II_ and K_II_` points are folded to a double Dirac cone at *Γ* point in the BZ center when taking the larger hexagonal unit cell (as displayed in Fig. 1d). The insets in Fig. 1d show the fourfold degenerated eigenstates with dipole and quadrupole modes. The relative parameters we use are *μ*_c1_ = 0.3 eV, *μ*_c2_ = 0.6 eV, and *τ* = 1 ps, which are moderately chosen from the previous researches for practical graphene [32, 33].

**Fig. 2:**
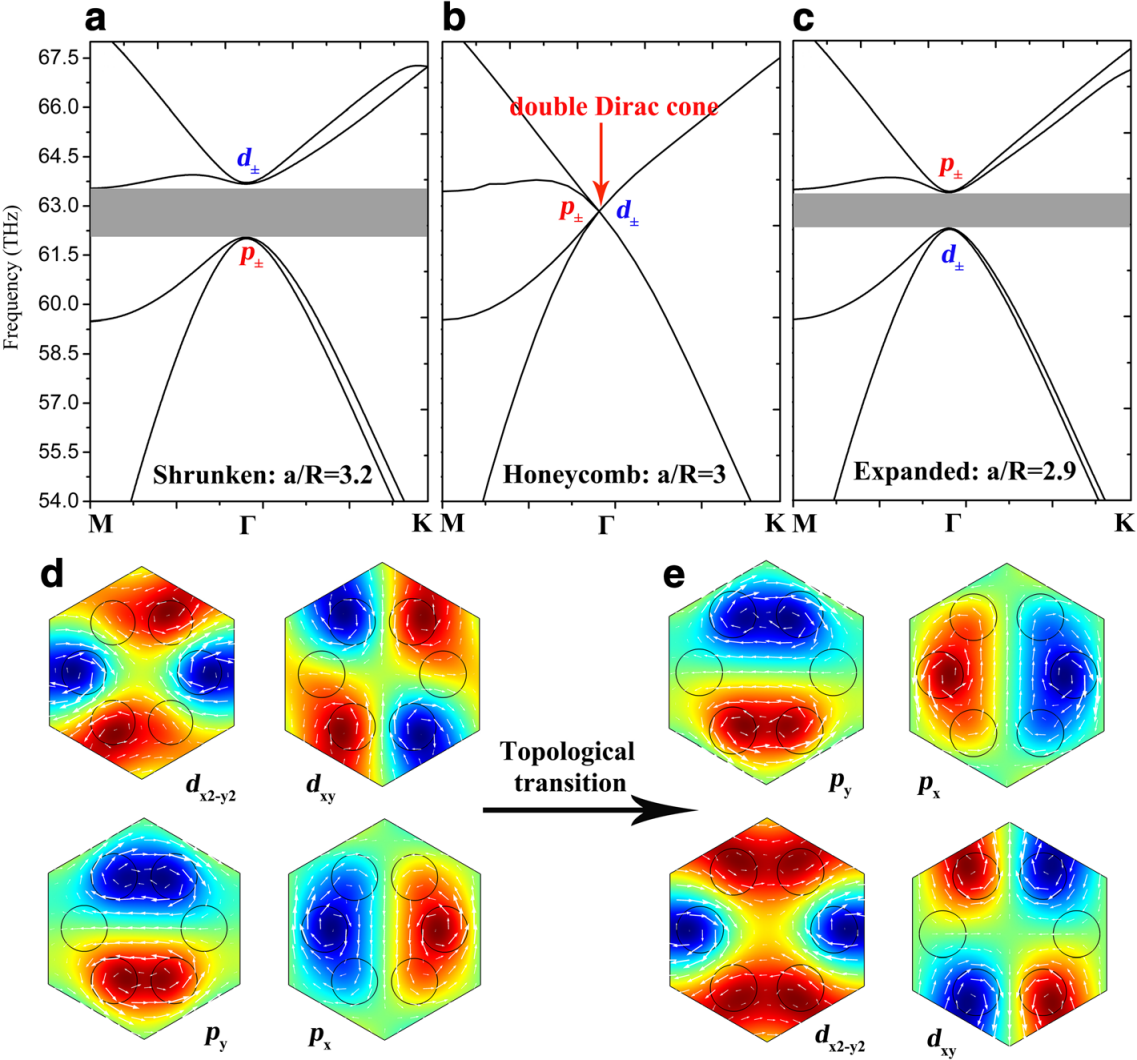
Band structures of the GPCs with **a** *a*/*R* = 3.2, **b** *a*/*R* = 3, and **c** *a*/*R* = 2.9. **d**, **e** The *E*_z_ field distributions of dipole modes and quadrupole modes of the *p*_±_ and *d*_±_ states in **a** and **c** respectively. The white arrows present the in-plane magnetic field associated with *E*_z_ field

The fourfold degenerated double Dirac cones composed of two dipolar and two quadrupolar modes are associated with two 2D irreducible representations of a C_6v_ point group, namely, E_1_ modes of odd spatial parity and E_2_ modes of even spatial parity. Following the conventional notation widely adopted in quantum mechanics [34], we can classify these modes to the *p*_*x*_/*p*_*y*_ and *d*_*x2-y2*_/*d*_*xy*_ modes according to their eigen *E*_*z*_ field distributions shown in Fig. 2. Next, in order to open a nontrivial topological band gap at the *Γ* point, we take further modifications (i.e., deforming the honeycomb lattice of *a*/*R* = 3) on the hexagonal unit cell to break the symmetry. When shrinking the graphene nanodisks to *a*/*R* = 3.2, the fourfold degenerated double Dirac cone splits into two twofold degenerate states and a bulk band gap opened from 62.1 to 63.5 THz as shown in Fig. 2a. The *E*_*z*_ fields of the lower bands have a pair of dipole modes exhibiting *p*_*±*_ characters, while the upper bands have a pair of quadrupole modes exhibiting *d*_*±*_ characters around the *Γ* point, which is consistent with the classic photonic theory that the dipole modes must exhibit lower frequency than the higher order quadrupole modes. However, a band inversion takes place when expanding the graphene nanodisks to *a*/*R* = 2.9, i.e., the dipole modes rise above the quadrupole modes, which brings about the topological nontrivial band gap from 62.4 to 63.3 THz as shown in Fig. 2c. Figure 2d, e illustrates the process of topological transition between *p*_*±*_ and *d*_*±*_ states, and the in-plane magnetic fields associated with *p*_*±*_ and *d*_*±*_ are marked with white arrows. The angular momenta of the wave function of *E*_*z*_fields *p*_*±*_ = (*p*_*x*_ ± i*p*_*y*_)/$\sqrt{2}$ and *d*_*±*_ = (*d*_*x2-y2*_ ± i*d*_*xy*_)/$\sqrt{2}$ further constitute the pseudospin in the present plasmonic crystals [17, 18].

To further explore the topological property of the band gaps shown in Fig. 2a, c, it is generally related to an effective Hamiltonian description and topological numbers. By applying the $\overset{\rightharpoonup }{k}\cdot \overset{\rightharpoonup }{p}$ perturbation theory, the effective Hamiltonian *H*_eff_(*k*) around the *Γ* point on the basis [*p*_*+*_*, d*_*+*_*, p*_*−*_*, d*_*−*_] can be expressed as [17, 35].

5 $$ {H}^{\mathrm{eff}}(k)=\left[\begin{array}{cccc}M+{Bk}^2& {Ak}_{+}& 0& 0\\ {}{A}^{\ast }{k}_{-}& -M-{Bk}^2& 0& 0\\ {}0& 0& M+{Bk}^2& {Ak}_{-}\\ {}0& 0& {A}^{\ast }{k}_{+}& -M-{Bk}^2\end{array}\right], $$

where *k*_*±*_ = *k*_*x*_ ± i*k*_*y*_, and *A* comes from off-diagonal elements of the first-order perturbation term ${M}_{\alpha \beta}=\left\langle {\Gamma}_{\alpha}\left|\overset{\rightharpoonup }{k}\cdot \overset{\rightharpoonup }{p}\right|{\Gamma}_{\beta}\right\rangle$ with *α* = 1, 2 and *β* = 3, 4. The effective Hamiltonian *H*_eff_(*k*) takes a similar form as the Bernevig-Hughes-Zhang (BHZ) model for the CdTe/HgTe/CdTe quantum well system [36], implying a topological band gap when the band inversion takes place. Based on the Hamiltonian expressed in Eq. (5), we can evaluate the spin Chern numbers of the topological plasmonic crystals as [36].

6 $$ {C}_{\pm }=\pm \frac{1}{2}\left[\operatorname{sgn}(M)+\operatorname{sgn}\left(-B\right)\right]. $$

Here, *M* = (*E*_*p*_ – *E*_*d*_)/2 is the frequency difference between *E*_*2*_ and *E*_*1*_ representations at the *Γ* point. *B* is determined by the diagonal elements of the second-order perturbation term and is typically negative [19]. Thus, *C*_*±*_ = 0 is obtained when having a normal band order as displayed in Fig. 2a. And we conclude that the band gap opened is trivial. However, *M* becomes positive when band inversion occurs. Therefore, *C*_*±*_ = ±1 is simply obtained, and the gap in Fig. 2c is nontrivial.

**Fig. 3:**
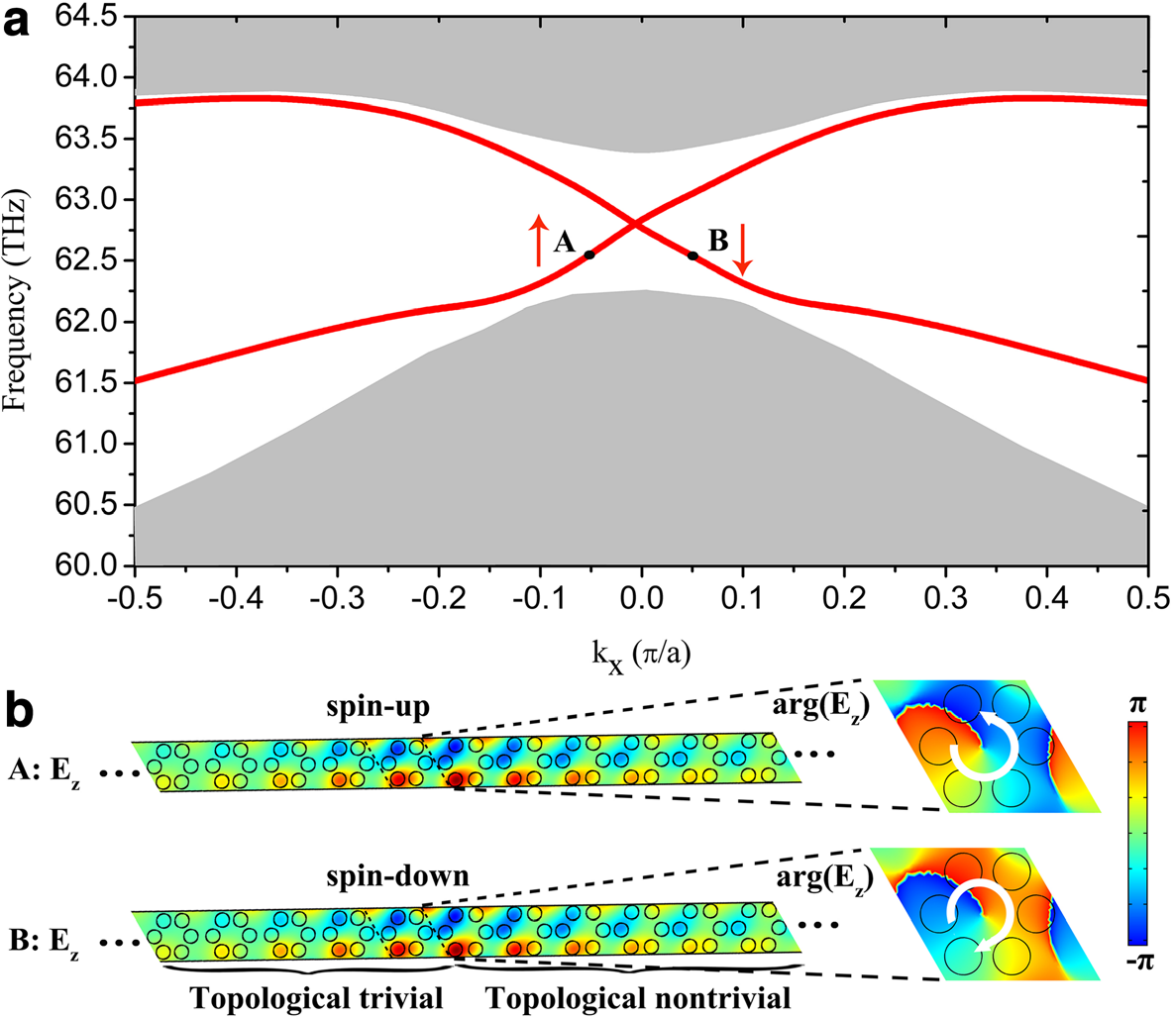
**a** Projected band structure for a supercell composed of 16 nontrivial unit cells cladded by 12 trivial unit cells on both sides. **b** Electric field distributions around the interface between the trivial and nontrivial plasmonic crystals at points A and B, i.e., at *k*_*x*_ = − 0.05π/a and 0.05π/a respectively

By overlapping the band gaps with different topologies (i.e., topological trivial and topological nontrivial), one can create edge states that are spatially confined around the interface between two plasmonic crystals. Here, we consider a ribbon of topologically nontrivial plasmonic crystal (with band structure shown in Fig. 2c) with its two edges cladded by two topologically trivial plasmonic crystals (with band structure shown in Fig. 2a) at the same frequency window. The two trivial regions prevent possible edge states from leaking into free space. In Fig. 3a, we present the calculated projected band structures along the *Γ*K direction for such a ribbon, where a bulk band gap is spanned by additional topological edge states as indicated by the double degenerated red curves. Fig. 3b plots the electric field distributions confined around the interface constructed by two distinctive crystals, corresponding to points A (with *k*_*x*_ = − 0.05π/*a*) and B (with *kx* = 0.05π/*a*) marked in Fig. 3a. The pseudo spin-up and spin-down characteristics are evidenced by phase vortexes of counterclockwise and clockwise as illustrated on the right panel of Fig. 3b.

**Fig. 4:**
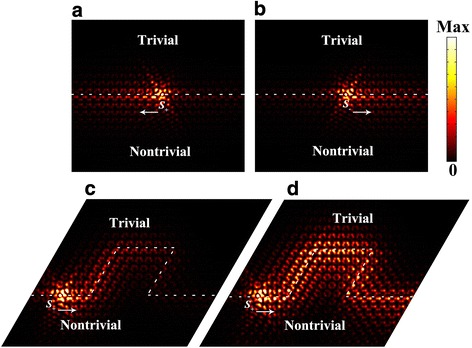
**a** Leftward and **b** rightward one-way edge states excited by in-plane magnetic field with a π/2 phase difference:${S}_{\pm }={H}_0\left(\overset{\rightharpoonup }{x}\mp i\overset{\rightharpoonup }{y}\right)$. **c** Topological edge states traveling along sharp bends. **d** The electric field intensity distribution of the topological one-way transmission without considering the intrinsic loss of graphene material

The pseudospin-dependent unidirectional transmission of edge states are also demonstrated in a finite 20*a* × 18*a* lattice constructed by the trivial and nontrivial crystals. As shown in Fig. 4a, b, one-way propagation of the SPP wave towards left (right) direction when excited by a pseudo spin-up (spin-down) source *S*_*+*_ (*S*_*−*_) of anticlockwise (clockwise) circular polarization of in-plane magnetic field. One of the most distinguishing features of topological edge states is that they are robust against perturbations/imperfections. To verify this robustness, we construct sharp bends as displayed in Fig. 4c, where the unidirectional transmission of SPP wave is excited by a pseudo spin-down source *S*_*−*_. The SPP wave vanished eventually after a long traveling distance along the sharp bends due to the intrinsic loss of graphene material. To further confirm this topological transmission, we also exhibit the electric field intensity distribution by ignoring the intrinsic loss of graphene for comparison. As can be seen from Fig. 4d, the SPP wave follows the designed route and maintains the one-way propagation with little backscattering.

## Conclusions

In summary, we have systematically investigated the band topologies of the GPCs constructed by periodically patterned graphene nanodisks. By employing zone folding mechanism, the Dirac cones at the BZ corner are folded to a double Dirac cone at the BZ center. Further, topological band gaps are realized by deforming the honeycomb lattice GPCs. Based on the effective Hamiltonian derived by the $\overset{\rightharpoonup }{k}\cdot \overset{\rightharpoonup }{p}$ perturbation theory, the spin Chern numbers are evaluated. The pseudospin characteristics, evidenced by phase vortexes of counterclockwise and clockwise, are successfully used to realize the unidirectional transmission of edge states along an interface constructed by two topological trivial and nontrivial plasmonic crystals. The designed GPC provides a new path to research topological phenomena and may find potential applications in the fields of topological plasmonics. It might also trigger the exploration of the pseudospin plasmonics and the technique of the pseudospin multiplexing in high-density nanophotonic integrated circuits.

## Abbreviations

BHZ: Bernevig-Hughes-Zhang
BZ: Brillouin zone
FEM: Finite element method
GPC: Graphene plasmonic crystal
QHE: Quantum Hall effect
QSHE: Quantum spin Hall effect
SPPs: Surface plasmon polaritons

## References

[1] He C, Sun XC, Liu XP, Lu MH, Chen Y, Feng L, Chen YF (2016). Photonic topological insulator with broken time-reversal symmetry. Proc Natl Acad Sci U S A.

[2] Khanikaev AB, Mousavi SH, Tse WK, Kargarian M, Macdonald AH, Shvets G (2013). Photonic topological insulators, Nat. Mater.

[3] Jörg C, Letscher F, Fleischhauer M, Freymann GV (2017). Dynamic defects in photonic Floquet topological insulators. New J Phys.

[4] Cheng X, Jouvaud C, Ni X, Mousavi SH, Genack AZ, Khanikaev AB (2016). Robust reconfigurable electromagnetic pathways within a photonic topological insulator. Nat Mater.

[5] Hafezi M (2014). Measuring topological invariants in photonic systems. Phys Rev Lett.

[6] Hatsugai Y (1993). Chern number and edge states in the integer quantum Hall effect. Phys Rev Lett.

[7] Chen ZG, Mei J, Sun XC, Zhang X, Zhao J, Wu Y (2017). Multiple topological phase transitions in a gyromagnetic photonic crystal. Phys Rev A.

[8] Fang K, Yu Z, Fan S (2012). Realizing effective magnetic field for photons by controlling the phase of dynamic modulation. Nat Photonics.

[9] Yang B, Wu T, Zhang X (2017). Engineering topological edge states in two dimensional magnetic photonic crystal. Appl Phys Lett.

[10] Ma T, Khanikaev AB, Mousavi SH, Shvets G (2015). Guiding electromagnetic waves around sharp corners: topologically protected photonic transport in metawaveguides. Phys Rev Lett.

[11] Dong JW, Chen XD, Zhu H, Wang Y, Zhang X (2017). Valley photonic crystals for control of spin and topology. Nat Mater.

[12] Hafezi M, Mittal S, Fan J, Migdall A, Taylor JM (2013). Imaging topological edge states in silicon photonics. Nat Photonics.

[13] Hafezi M, Demler EA, Lukin MD, Taylor JM (2012). Robust optical delay lines with topological protection. Nat Phys.

[14] Maczewsky LJ, Zeuner JM, Nolte S, Szameit A (2017). Observation of photonic anomalous Floquet topological insulators. Nat Commun.

[15] Rechtsman MC, Zeuner JM, Plotnik Y, Lumer Y, Podolsky D, Dreisow F, Nolte S, Segev M, Szameit A (2013). Photonic Floquet topological insulators. Nature.

[16] Barik S, Miyake H, Degottardi W, Waks E, Hafezi M (2016). Two-dimensionally confined topological edge states in photonic crystals. New J Phys.

[17] Wu LH, Hu X (2015). Scheme for achieving a topological photonic crystal by using dielectric material. Phys Rev Lett.

[18] He C, Ni X, Ge H, Sun XC, Chen YB, Lu MH, Liu XP, Chen YF (2016). Acoustic topological insulator and robust one-way sound transport. Nat Phys.

[19] Mei J, Chen Z, Wu Y (2016). Pseudo-time-reversal symmetry and topological edge states in two-dimensional acoustic crystals. Sci Rep.

[20] Zhang Z, Wei Q, Cheng Y, Zhang T, Wu D, Liu X (2017). Topological creation of acoustic pseudospin multipoles in a flow-free symmetry-broken metamaterial lattice. Phys Rev Lett.

[21] Barnes WL, Dereux A, Ebbesen TW (2003). Surface plasmon subwavelength optics. Nature.

[22] Iurov A, Huang D, Gumbs G, Pan W, Maradudin A (2017). Effects of optical polarization on hybridization of radiative and evanescent field modes. Phys Rev B.

[23] Memmi H, Benson O, Sadofev S, Kalusniak S (2017). Strong coupling between surface plasmon polaritons and molecular vibrations. Phys Rev Lett.

[24] Fei Z, Rodin A, Andreev G, Bao W, McLeod A, Wagner M, Zhang L, Zhao Z, Thiemens M, Dominguez G (2012). Gate-tuning of graphene plasmons revealed by infrared nano-imaging. Nature.

[25] Chen J, Badioli M, Alonso-González P, Thongrattanasiri S, Huth F, Osmond J, Spasenović M, Centeno A, Pesquera A, Godignon P (2012). Optical nano-imaging of gate-tunable graphene plasmons. Nature.

[26] Vakil V, Engheta N (2011). Transformation optics using graphene. Science.

[27] Jin D, Christensen T, Soljačić M, Fang NX, Lu L, Zhang X (2017). Infrared topological plasmons in graphene. Phys Rev Lett.

[28] Pan D, Yu R, Xu H, FJG d A (2017). Topologically protected Dirac plasmons in a graphene superlattice. Nat Commun.

[29] Lu H, Zeng C, Zhang Q, Liu XM, Hossain M, Reineck P, Gu M (2015). Graphene-based active slow surface plasmon polaritons. Sci Rep.

[30] Low T, Avouris P (2014). Graphene plasmonics for terahertz to mid-infrared applications. ACS Nano.

[31] Jablan M, Buljan H, Soljačić M (2009). Plasmonics in graphene at infrared frequencies. Phys Rev B.

[32] Bolotin KI, Sikes K, Jiang Z, Klima M, Fudenberg G, Hone J, Kim P, Stormer H (2008). Ultrahigh electron mobility in suspended graphene. Solid State Commun.

[33] Efetov DK, Kim P (2010). Controlling electron-phonon interactions in graphene at ultrahigh carrier densities. Phys Rev Lett.

[34] Dresselhaus MS, Dresselhaus G, Jorio A (2007). Group theory: application to the physics of condensed matter (Springer Science & Business Media).

[35] Klipstein PC (2010). Operator ordering and interface-band mixing in the Kane-like Hamiltonian of lattice-matched semiconductor superlattices with abrupt interfaces. Phys Rev B.

[36] Bernevig BA, Hughes TL, Zhang SC (2006). Quantum spin Hall effect and topological phase transition in HgTe quantum wells. Science.

